# A Pilot Study on the Relationship between Obstructive Sleep Apnoea–Hypopnea Syndrome, Neurodevelopment, and Ricketts’ Cephalometry

**DOI:** 10.3390/jcm13175274

**Published:** 2024-09-05

**Authors:** Teresa I. González Robayna, Carlos Pérez-Albacete Martínez, Jesús M. Gandía, Mª Dolores Austro Martínez, Ángela Sempere Pérez, Raúl Ferrando Cascales

**Affiliations:** 1 UCAM Faculty of Dentistry, University Campus Los Jerónimos, Catholic University of Murcia, 135 Guadalupe, 30107 Murcia, Spain; cperezalbacete@ucam.edu (C.P.-A.M.); rferrando@ucam.edu (R.F.C.); 2 Department of Mathematics, Physics and Technological Sciences, CEU University Cardenal Herrera, 03202 Elche, Spain; jesus.martinezgandia@uchceu.es; 3 Statistic, Mathematics and IT Department, University Miguel Hernández, 03202 Elche, Spain; 4 Dentistry Department, CEU University Cardenal Herrera, 03202 Elche, Spain; maria.austromartinez@uchceu.es; 5 HLA Vistahermosa Clinic Avenida de Dénia, 103, 03015 Alicante, Spain; udac@ctielx.es

**Keywords:** OSAHS, VERT index, cephalometry, neurodevelopment, facial growth, sleep disorders

## Abstract

**Background**: The aim of this research is to achieve the early detection of facial characteristics in patients diagnosed with neurodevelopmental deficits and obstructive sleep apnoea–hypopnea syndrome (OSAHS) through the analysis of the VERT index and Ricketts’ cephalometry to minimise the neurocognitive morbidity associated with these disorders. **Methods**: This clinical study was conducted on 44 patients aged 4 to 15 years. Participants completed an initial questionnaire about sleep disturbances, followed by a polysomnography, a radiographic study, and an oral examination. **Results**: The maximum variability in the data was obtained in the mandibular plane angle, where we observed that the measurement was higher in patients diagnosed with OSAHS. The relative and normalised indices of facial depth and the mandibular plane showed variations between patients with a clinical picture compatible with OSAHS and the control group without pathology. **Conclusions**: Our findings indicate that Ricketts’ VERT index by itself is unable to provide evidence of a diagnosis compatible with OSAHS in patients with early neurodevelopmental deficits, but, after analysing the results obtained, we observed that for the cephalometric measurements of the mandibular plane angle and facial depth relative to the patient’s age, there are sufficiently strong variations for creating a solid method of differential diagnosis, thus preventing complications at the neurocognitive level.

## 1. Introduction

According to the World Health Organisation, neurodevelopmental disorders affect one in six children in industrialised countries [1]. Disorders affecting brain growth and development cover a wide spectrum of pathologies, such as intellectual disability, Tourette’s syndrome, autism spectrum disorder, learning disabilities, and attention deficit disorder with or without hyperactivity (ADD/ADHD) [2]. The relationship between sleep and neurodevelopmental disorders conforms a wide field of study in which there are a large number of factors to be taken into account, which makes it difficult to approach from a single point of view. Primary sleep disorders, such as sleep apnoea–hypopnea syndrome (AHI), restless legs syndrome (RLS), periodic limb movement disorder (PLMD), and narcolepsy, can cause similar symptomatology to attention deficit hyperactivity disorder (ADHD), such as inattention, hyperactivity, and disruptive behaviours due to poor impulse control in children with these disorders [3]. Similarly, in children with sleep breathing problems, the frequency of behavioural disturbances and attention problems is three times higher [4]. Each episode of apnoea may be accompanied by a period of hypoxia, which increases the risk of chronic sleep deprivation and the development of OSAHS-related complications such as growth retardation, pulmonary and/or systemic hypertension, cardiac dysfunction, systemic inflammation, neurocognitive impairment, learning and behavioural problems, and a decrease in the quality of life [5]. Different authors have created various specific questionnaires [6,7], such as the BISQ, BEARS, or SDSC (Bruni) questionnaires, focused on the diagnosis of sleep disorders, although the questionnaires per se are insufficient for the diagnosis of sleep disorders. Medical history, otorhinolaryngologic examination, and snoring audiotapes have been shown to have low sensitivity and specificity for diagnosis and limited usefulness, as they are indicative of OSAHS if positive, but they have a high false negative rate. No combination of medical history and physical examination findings has the discriminatory ability to diagnose or rule out the presence of OSAHS [8]. Overnight assisted polysomnography (PSG) is the accepted gold standard for the diagnosis of paediatric OSAHS [9]. PSG records, in a standardised and simultaneous way, multiple biological signals during the sleep–wake states, performing a functional evaluation that complements the parameters of brain function (electroencephalogram, EEG), respiratory, vital, and movement signs, which allow us to evaluate the progression of the sleep cycle and rule out respiratory disorders, abnormal limb movements, and paroxysmal epileptic or non-epileptic events, among other causes [10,11].

The neurocognitive morbidity associated with sleep disorders may only be partially reversible, with the degree of hypoxaemia correlating with deficits in executive function, while the intensity of sleep fragmentation would be associated with impaired attention [12]. It is important to note that a delay in the diagnosis and treatment of OSAHS may result in the non-complete reversibility of the impairments associated with the syndrome, increasing the need for a diagnosis that addresses the condition at an early stage [13,14]. There is a relationship between patients with neurodevelopmental deficits and breathing problems during sleep, resulting in a predominant mouth breathing pattern, which is associated with recurrent cephalometric values of certain facial biotypes.

The concept of facial biotype was described by Ricketts, defining it as the set of morphological and functional characteristics that determine the direction of growth and the shape of the face, being essential to distinguish the different growth patterns and predict the rotation of maxillofacial growth, necessary for the diagnosis and treatment of our patients [15,16,17].

Ricketts’ VERT index defines the facial biotype of the patient. In the context of Ricketts’ analysis, VERT is an acronym that stands for “vertical”. It refers to a composite index that Ricketts created to assess the vertical growth pattern of the face. This index is the result of a combination of several cephalometric measurements that determine the direction of facial growth (more vertical or more horizontal).

Ricketts’ VERT index defines the patient’s facial biotype. It represents a coefficient of variation that numerically establishes the type and amount of vertical growth in the lower third of the face caused by the posterior or anterior rotation of the mandible [15]. This index evaluates the difference between a patient’s measurements at various facial angles and the established norms. A negative sign is assigned if the measurement deviates towards dolichocephaly, a positive sign if it deviates towards brachycephaly, and zero if it is within the norm. The difference between the patient’s measurement and the norm is obtained and divided by the standard deviation of the measurement analysed. These values are added algebraically and divided by the number of factors under study (five in this case). The result obtained is the patient’s VERT index, which is then compared with the reference values established by Ricketts.

However, the calculation of the VERT index can be conducted using the standard established for 9-year-old patients or, for greater accuracy, by customising the standard according to the patient’s age. This approach is justified because three of the five factors mentioned above change with age. Therefore, Ricketts suggests an age adjustment table (Table 1). Factors that vary with age are facial depth, mandibular plane angle, and mandibular arch. Age adjustment is applied for females up to 14 years and for males up to 16 years, at which age growth is considered to be virtually complete [16,17]. We hypothesise that there is a relationship between patients with neurodevelopmental deficits and breathing problems during sleep, resulting in a predominant mouth breathing pattern, which is associated with recurrent cephalometric values of certain facial biotypes.

## 2. Materials and Methods

### 2.1. Study Subjects

This clinical study was conducted on 44 patients aged between 4 and 15 years in Interfamily Therapy Centres in the city of Elche (Alicante), from 2021 to 2024. Initially, participants were given a screening questionnaire on sleep disturbances, which was completed by the parents or guardians of the participants. An intervention group of 19 patients diagnosed with neurodevelopmental deficits and a control group of 25 healthy patients were formed. The healthy patients in this study were patients who underwent polysomnography for reasons unrelated to OSAHS, such as headaches, epilepsy, and sleep disturbances. The patient selection criteria in our study included patients diagnosed with neurodevelopmental deficits, patients aged between 4 and 15 years, patients without previous OSAHS treatment, patients without associated pathologies, and patients not previously treated with orthodontics. Among the exclusion criteria, patients older than 15 years with associated respiratory pathologies or other syndromes, patients younger than 4 years, patients undergoing adenotonsillectomy surgery, and patients with inflammatory respiratory diseases such as asthma or allergic rhinitis were excluded as these could make it difficult for air to enter or worsen the symptoms. The statistical data on the age of the patients differentiated by diagnosis are shown in Table 2.

### 2.2. Data Collection and Analysis

All study subjects were evaluated by paediatricians, neurophysiologists, and technical specialists and subjected to the specific diagnostic questionnaires ADI-R (Autism Diagnostic Interview—Revised); DISCO (Diagnosis Interview for Social and Communication Disorder); ADOS-G (Autism Diagnostic Observation Schedule—Generic); and M-CHAT (Modified Checklist for Autism in Toddlers) [18,19,20], and they were subjected to the clinical judgement of experienced neuropaediatricians associated with the medical centre. The results of these tests were interpreted by a neuropaediatrician to establish a diagnosis of a neurodevelopmental disorder. Patients in the control group were submitted to a PSG for reasons unrelated to OSAHS, such as epilepsy, narcolepsy, periodic limb movement disorder, unusual sleep behaviours or parasomnias, or chronic unexplained insomnia. All parents or legal guardians of the study participants were provided with a questionnaire on the sleep habits of these children: the Bruni Sleep Disturbance Scale for Children (SDSC). The SDSC consists of 27 items rated on a Likert-type scale and is designed to detect sleep disorders divided into six categories, problems in initiating or maintaining sleep, breathing problems, awakening disorders, sleep–wake transition disturbances, excessive daytime sleepiness, and nocturnal hyperhidrosis in children aged between 6 and 15 years, when aspects such as daytime sleepiness, breathing problems, sleep–wake transition disturbances, and more are explored (Table 3). The cut-off point for global sleep disorder is 39 [21]. Patients with SDSC test scores above 39 were referred to a hospital for PSG, which were performed with Nicolet Natus Sleep Works v.44 polysomnography equipment.

**Figure 1:**
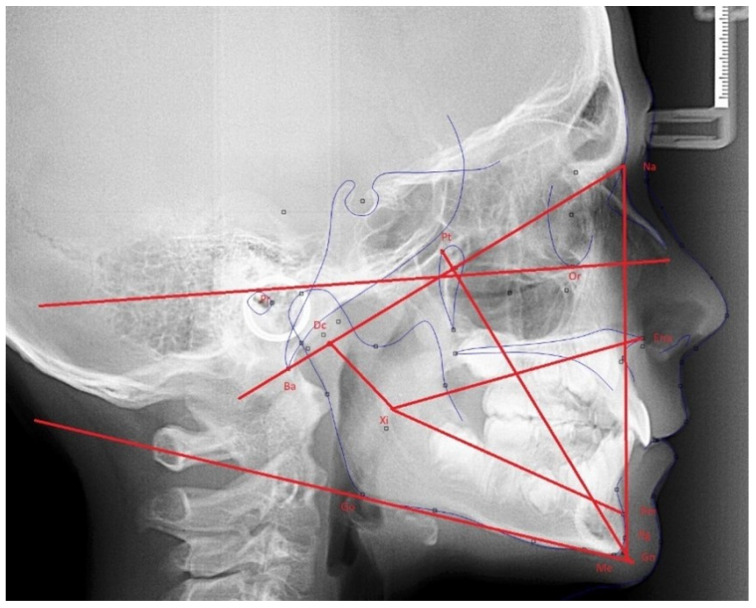
VERT-dependent cephalometric measurements.

Subsequently, all patients participating in this study were scheduled for oral, facial, and radiographic examination, where facial and cephalometric features were taken into consideration for the subsequent Ricketts analysis (Figure 1). Lateral cephalometric radiographs were obtained for each subject with the head in the natural position and the teeth in maximum intercuspidation [22,23,24]. These lateral cephalometric radiographs were digitally measured by the same investigator (the first author of this study) using Gesden G5 version 5.43 and Ortomed software version 4.5.

### 2.3. Data Collection for Ricketts’ VERT Index and Related Measures

Once the data described above were obtained, the patients were scheduled for an oral, facial, and radiographic examination, in which the facial and cephalometric characteristics were taken into consideration, as well as for the collection of all the data from these tests for subsequent statistical analysis. The resulting VERT index measures, as well as all cephalometric measures dependent on this index, were recorded to study statistical deviations between the groups for these measures independently.

## 3. Results

This study tested whether Ricketts’ VERT index showed significant differences between the groups differentiated by OSAHS diagnosis. Given the number of subjects in each group (*n* < 30), normality tests (Shapiro–Wilk) were performed on both groups, showing a normal distribution (*p*-value = 0.632, 0.916) and allowing the statistical analysis to continue. These values are shown in Table 4 as well as the descriptive values of each group for this index.

**Figure 2:**
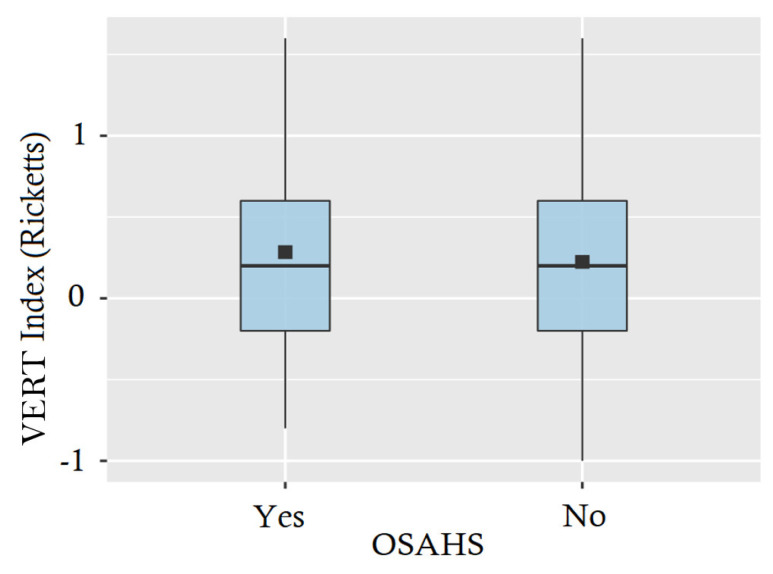
A descriptive box plot of the Ricketts’s VERT index measurements categorised by OSA diagnosis. The lack of diagnostic differentiation is evident due to the uniformity of measurements across groups.

According to these results, Student’s *t*-test (significance 0.05) was performed on both groups, which concluded that there were no significant differences between patients with and without a diagnosis of OSAHS in the values of Ricketts’ VERT index (*p*-value = 0.748, CI (95%) = [0.317, 0.417]), so it cannot be concluded that the VERT index is in itself a predictive parameter of the existence of OSAHS (Figure 2).

On the other hand, measures related to the age of the patients were obtained, emphasising the deviation in the measures with regard to the normalised cephalometric measure according to Ricketts’ studies. We must therefore remember that, from now on, statistical contrasts will be carried out with respect to these measurements; for example, in the case of the mandibular plane, the result would be as follows:

MP_rel_ = |MP − MP_norm_|

where MP is the measurement of the subject’s mandibular plane, and MP_norm_ is the normalised measurement to the patient’s age, which is given in Table 5. In the same manner as the VERT index, necessary statistical tests were carried out to be able to implement the contrasts independently, obtaining normality in each of them individually, and the descriptive results are shown in Table 4.

For this reason, to ensure the validity of the analyses conducted, thorough checks for normality were performed using the Shapiro–Wilk test for each variable studied. The results of these tests, along with their corresponding *p*-values, are detailed in Table 4. Additionally, the homogeneity of variances was assessed through tests for homoscedasticity. The results indicated that the assumption of homogeneity of variances was not met. Due to this violation of homoscedasticity, Welch’s *t*-test was applied to the contrasts carried out on the cephalometric measurements, as this test appropriately adjusts for the lack of variance homogeneity, providing more robust and reliable results.

As we can see in Table 6, there were significant differences for only two of the five cephalometric measures under study, which are the ones related to the facial depth *p*-value = 0.011, CI (95%) = [0.58, ∞), and that relative to the mandibular plane *p*-value < 0.001, CI (95%) = [1.42, ∞), so that the deviation from the normal for the age of the patients was significantly higher in these measures in patients diagnosed with OSAHS.

**Figure 3:**
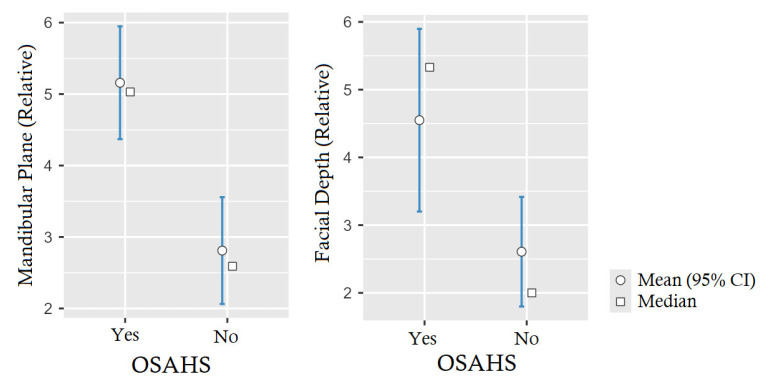
Graphic of 95% confidence interval of relative mandibular plane (*p*-value < 0.001) and relative facial depth (*p*-value = 0.011) differentiated by OSAHS.

It is important to note that the measures of the mandibular arch and lower facial height are different in each group; however, although this tendency can be seen, the data do not show that it is significantly possible to establish such a correlation (*p*-value = 0.222 and *p*-value = 0.164, respectively). The same occurs with the facial axis, where in this case, patients with a diagnosis of OSAHS presented a smaller angle than patients without such a diagnosis, opposing the trend of the rest of the measures, although again, we did not obtain a sufficient level of confidence to establish a hypothesis in this respect (*p*-value = 0.166), as can be seen in Table 6. Figure 3 shows the 95% confidence intervals obtained through the inference for each value, implementing the results described above.

## 4. Discussion

Craniofacial morphology plays an important role in OSAHS, with cephalometry being a fundamental value when analysing facial structures. According to our results, we consider that patients with larger deviations from standardised measurements in the mandibular plane and facial depth should undergo cephalometry to rule out OSAHS, although it should not be used as the sole diagnostic tool but as a complement to PSG.

In addition, during the craniofacial growth process, various factors can significantly affect the VERT index and cephalometric values, such as mandibular rotation, bone growth, and soft tissue modifications. The changes observed in this index during growth are closely linked to the developmental dynamics of facial bones and tissues. Therefore, it is extremely important to take these factors into account when diagnosing and providing possible treatments for patients with disorders related to craniofacial growth.

The current study of the morphology of our patients showed that only the VERT index had no predictive value for childhood OSAHS. Only an isolated number of studies have used the VERT index as a diagnostic tool combined with clinical assessment. Bozzini et al. [25] attempted to determine the clinical and anatomical features associated with the severity of OSAHS in children with tonsillar hypertrophy, finding no significant differences in the clinical criteria of the mild, moderate, or severe OSAHS groups, although like our results, they found significantly different variations in the facial depth angle. For Bozzini et al., the craniofacial characteristics did not influence the severity of the disease, which differs from our findings, because in their study, they excluded pubertal patients, basing their sample on children aged between 4 and 9 years, where the full extent of skull growth was not expressed. Furthermore, there are important differences in the sample, as Bozzini et al. did not take into account patients with neurological disorders, which is important for us as not only are clinical parameters relevant but also anatomical parameters for the diagnostic process of our patients. By excluding these patients, they did not take into account the differences associated with neuromuscular factors that affect a large number of patients. Other authors [26] also excluded children with neurological syndromes and disorders from their study. They also used the VERT cephalometric analysis and concluded that the proportion of boys and girls affected by OSAHS was the same while finding variations in the mandibular plane angle and facial depth but only in boys and not in girls, differing from our study in which sex did not establish a significant difference. Knowing that the increase in the mandibular plane angle reflects the vertical direction of growth and that facial depth reflects the position of the mandible with regard to the skull base, Di Francesco et al. [26] demonstrated a positive association between sleep apnoea and the VERT index, indicating that the dolichofacial pattern may play an important role in the pathophysiology of sleep apnoea in children with high AHI, thus confirming what was observed in our study. However, an individualised study of the parameters that make up the VERT index was able to demonstrate significant differences in the diagnostic differentiation of OSAHS. The relative and normalised indices (see Methodology) of the facial depth and mandibular plane showed variations between patients with a clinical picture compatible with the syndrome and the control group; this corroborates what was already presented by Galeotti et al. [27], who not only found a correlation between the mandibular angle but also found discrepancies in the ANB angle and upper pharyngeal width. On the other hand, Liu et al. [28] found a relationship between the angle of the mandibular plane and OSAHS, in addition to an increase in mandibular protrusion and retrognathia, thus differing from Bozzini et al. [25]’s findings, which found no direct relationship between the craniofacial structure and the severity of OSAHS, although they did find a relationship in airway width, as also observed by Galeotti et al. [27], who related the reduction in nasopharyngeal width to the maxillomandibular hyperdivergent growth pattern, supporting the correlation between OSAHS and craniofacial characteristics. Similarly, Hasanin et al. [29] observed a narrower airway in ADHD patients, without finding differences in craniofacial characteristics and the growth pattern as we found in our study. The maximum variability in the data obtained in our research was observed in the angle of the mandibular plane, where we observed that the angle is significantly higher in these measurements in patients diagnosed with OSAHS, confirming the findings of Deng Gao et al., Feng et al., Wang et al., [30,31,32], and Lowe et al., [33], who already analysed and found differences in the skeletal, dental, and soft tissue components of the craniofacial structure of patients that predispose them to suffer from OSAHS. We were unable to find significant differences between healthy patients and those with OSAHS and neurodevelopmental deficits related to the parameter for lower facial height as found by other authors [6,34,35], which for them supported the relationship between facial disharmony and OSAHS. We did not observe variability in the mandibular arch parameter between the control group and the neurodevelopmental deficit/OSAHS group, similar to Di Francesco et al. [26], who only found variability in children with high AHI values, with the dolichofacial pattern predominating.

## 5. Conclusions

Our findings indicate that Ricketts’ VERT index by itself is unable to provide evidence of a diagnosis compatible with OSAHS in patients with early neurodevelopmental deficits. For the cephalometric measurements of the mandibular plane angle and facial depth relative to the patient’s age, there are sufficiently strong variations for creating a solid method of differential diagnosis by applying the formula presented in Section 2.2.

Early diagnosis and intervention through the calculation of the angle of the mandibular plane and facial depth would minimise the adverse effects of OSA at the neurocognitive level, thus preventing medium- and long-term complications such as learning difficulties and cardiovascular complications. In this way, we can draw up specific prevention strategies and avoid unnecessary pharmacological treatments as a consequence of the high comorbidity presented between neurodevelopmental disorders and OSA.

## 6. Limitations

There are some limitations to this pilot study, particularly around the variability in the results. This variability stems from the following: the different study designs, the diagnostic protocols used, and the small sample size. The limited sample size was partly due to the challenges faced by participants in undergoing polysomnography; this sample size impacted this study’s statistical power. Despite these challenges, the inferential analyses conducted demonstrate the diagnostic differentiation capability of the results, confirming their reliability. Based on the results, future studies with larger samples will be necessary, as well as an evaluation of the presence and severity of the hypertrophy of the lymphoid tissue of the pharynx and nasopharynx.

## Figures and Tables

**Table 1:** Ricketts VERT index rating. DCP: Doliocephaly, MCP: Mesocephalic, BCP: brachycephaly.

Severe DCP	DCP	Soft DCP	MCP	BCP	Severe BCP
−2	−1	−0.5	0	+0.5	+1

**Table 2:** Age of study subjects differentiated by OSAHS diagnosis.

OSAHS	N	Mean	Median	SD	Minimum	Maximum
Yes	19	8.73	9	2.79	5	14
No	25	10.04	10	2.26	5	14

**Table 3:** Study subjects with and without the disease.

Neurodevelopmental Disorder			
OSAHS	Yes	No	Total
Yes	19	0	19
No	3	22	25
Total	22	22	44

**Table 4:** Descriptive statistics on cephalometric measurements differentiated by diagnosis of OSAHS.

Cephalometric Measurements							Shapiro–Wilk	
	OSAHS	N	Mean	SD	Minimum	Maximum	W	*p*
Mandibular plane (Relative)	Yes	19	5.16	1.76	1.92	7.79	0.94	0.319
	No	25	2.81	1.91	0.06	7.42	0.95	0.238
Facial depth (Relative)	Yes	19	4.55	3.00	0.35	9.95	0.93	0.162
	No	25	2.61	2.06	0.08	8.94	0.85	0.001
Mandibular arch (Relative)	Yes	19	9.49	5.15	3.35	22.68	0.90	0.059
	No	25	8.24	5.56	0.75	20.64	0.92	0.064
Facial axis (Relative)	Yes	19	3.59	3.11	0.06	8.58	0.88	0.019
	No	25	4.51	3.08	0.69	12.70	0.93	0.103
Lower facial height (Relative)	Yes	19	5.04	3.49	1.17	13.12	0.89	0.036
	No	25	4.07	2.77	0.02	9.96	0.96	0.396
VERT index	Yes	19	0.28	0.63	−0.80	1.60	0.96	0.632
	No	25	0.22	0.60	−1.00	1.60	0.98	0.920

**Table 5:** Standardisation of orthodontic measurements.

AGE (Years)	9	10	11	12	13	14	15	16
Facial Axis	90°	90°	90°	90°	90°	90°	90°	90°
Facial Depth	87°	87.3°	87.6°	87.9°	88.2°	88.5°	88.8°	89.1°
Mandibular Plane	26°	25.7°	25.4°	25.1°	24.8°	24.5°	24.2°	23.9°
Facial Height	47°	47°	47°	47°	47°	47°	47°	47°
Mandibular Arch	26°	26.5°	27°	27.5°	28°	28.5°	29°	29.5°

**Table 6:** Results of mean comparison for cephalometric measurements and VERT index (Student’s *t*-test with Welch’s correction, significance 0.05).

					95% Confidence Interval	
Cephalometric Measurements	Statistical	*d f*	*p*-Value	Mean Difference	Lower	Upper
Facial depth (Relative)	2.42	30.34	0.011	1.94	0.58	∞
Mandibular plane (Relative)	4.24	40.40	<0.001	2.35	1.42	∞
Mandibular arch (Relative)	0.77	40.30	0.222	1.25	−1.48	∞
Lower facial height (Relative)	0.99	33.62	0.164	0.97	−0.68	∞
VERT index	0.32	42.00	0.749	0.06	−0.32	0.44
Facial axis (Relative)	−0.98	42.00	0.166	−0.92	−∞	0.66

## Data Availability

The data are unavailable due to privacy and ethical restrictions.
